# Prevalence of sleep disturbances in Chinese adolescents: A systematic review and meta-analysis

**DOI:** 10.1371/journal.pone.0247333

**Published:** 2021-03-04

**Authors:** Mengjiao Liang, Ling Guo, Jing Huo, Guoliang Zhou

**Affiliations:** 1 Department of Psychology, Education College, Jiujiang University, Jiujiang, Jiangxi Province, China; 2 Department of Special Education, College of Special Education, Leshan Normal University, Leshan, Sichuan Province, China; 3 Department of English Learning, School of Foreign Language, Northeast Forestry University, Harbin, Heilongjiang Province, China; 4 Department of Lifelong Learning, College of Lifelong Learning, Jiujiang University, Jiujiang, Jiangxi Province, China; Weill Cornell Medical College in Qatar, QATAR

## Abstract

**Objectives:**

To review cross-sectional studies on the prevalence of sleep disturbance in Chinese adolescents and use a meta-analysis to explore the factors that may explain the heterogeneity between estimates of the prevalence.

**Methods:**

We followed the Preferred Reporting Items for Systematic Review and Meta-analysis and searched the PubMed, Embase, Cochrane Library, PsycINFO, Scopus, Web of Science, SinoMed, Chinese National Knowledge Infrastructure (CNKI), WanFang, and VIP Database for Chinese Technical Periodicals databases from their inception through June 30, 2020. Analysis of the abstract, full-text, and data were conducted independently with uniform standards. Sub-group analyses and meta-regression analyses were performed to explore the associations between prevalence and gender, sex ratio, mean age, area, studying stage, sample size, survey time, response rate, assessment tools, PSQI cut-off, and quality score of the study.

**Results:**

A total of 63 studies (64 groups of outcomes) were included in our analysis, covering 430,422 adolescents across China, of which 104,802 adolescents had sleep disturbances. The overall pooled prevalence was 26% (95% CI: 24–27%). Adolescents in senior high school (28%, 95% CI: 24–31%, *p*<0.001) had a higher prevalence of sleep disturbances than those in junior high school (20%, 95% CI: 15–24%, *p*<0.001). Studies with effective sample size of more than 1,000 and less than 3,000 had the highest prevalence of 30% (95% CI: 24–35%, *p*<0.001). The prevalence of sleep disturbances was not affected by other factors.

**Conclusion:**

This systematic review and meta-analysis revealed that sleep disturbances are common in Chinese adolescents, and effective psychological and behavior intervention may be needed to help adolescents solve their sleep problems.

## Introduction

In recent years, sleep disturbances have become increasingly more common worldwide. According to the China Sleep Quality Survey Report in 2018, among the 100,000 respondents, 83.81% suffered from some type of sleep disturbance [1]. Sleep disturbances like insufficient sleep time, irregular sleep patterns, poor sleep quality, and daytime sleepiness are common in Chinese adolescents, due to the competitiveness of top-level educational resources and intense social pressure [2, 3]. Furthermore, the early start time for school, heavy coursework loads, evening and night social activities, and online game addiction accelerates the sleep debt of Chinese adolescents. However, poor sleep quality would lead to mood disorders, such as negative affect, anxiety, and depression, poor academic performance, cognitive impairment, obesity, and high blood pressure [4–8].

Several studies have assessed the prevalence of sleep disturbances in Chinese adolescents. However, the prevalence estimates have varied substantially, reflecting the differences in the characteristics of adolescents and assessment instruments. Thus, a systematic review and meta-analysis should be done to better understand the prevalence of sleep disturbances in Chinese adolescents to develop effective psychological and behavior intervention that may be needed to help adolescents solve their sleep problems.

Therefore, our aim was to estimate the prevalence of sleep disturbances in Chinese adolescents and explore the heterogeneity between studies using subgroup analyses and meta-regression analyses, in terms of characteristics, assessment tools, and study quality.

## Methods

This study is approved by the Ethics Committee of Medicine School of Jiujiang University with approval number JJU202006026.

### Search strategy

This systematic review and meta-analysis were performed according to the Preferred Reporting Items for Systematic Review and Meta-analysis (PRISMA) [9]. We searched the PubMed, Embase, Cochrane Library, PsycINFO, Scopus, Web of Science, SinoMed, Chinese National Knowledge Infrastructure (CNKI), WanFang, and VIP Database for Chinese Technical Periodicals databases from its inception to June 30, 2020, without language restrictions. The search strategy was approved by experts in sleep problems from the Department of Clinical Psychology and the Department of Neurology in hospital. Full search terms are provided in the Appendix in S1 File, including prevalence, epidemiology, cross-sectional study, rate in conjunction with sleep disturbances, insomnia, sleep disorders, sleep symptoms, sleep quality, and sleep problems in Chinese adolescents. The references of selected papers were screened in NoteExpress (Version 3.2.0) [10].

### Study selection

Studies were included in the analysis if they met the following criteria: (1) subjects aged from 13 to 19 years old in China; (2) cross-sectional epidemiology survey reported the prevalence of sleep disturbances with standardized assessment tools (Pittsburgh Sleep Quality Index/Athens Insomnia Scale/ICSD-2); (3) sample size ≥300; (4) full text written in Chinese or English. Studies were excluded for the following reasons: (1) only research articles were considered (conference abstracts, protocols, reviews, meta-analyses, citations, comments, and news release were excluded); (2) missing sampling method, sample size, or response rate; (3) based on experiments of clinical treatment or intervention; (4) focused on special population, such as patients with mental problems, students with post-traumatic stress disorder, and teenagers with medical conditions. Any disagreement in the screening procedure was discussed and resolved by a third reviewer.

### Data extraction and study quality

Two reviewers independently screened the titles, abstracts, and full text. A duplicate extraction form was used to record information, which was tabulated using Microsoft Excel. The information included survey year, geographic location (area), effective sample size, response rate, sampling methods, male proportion, age, studying stage, assessment instruments, the cut-off for sleep disturbance, prevalence of sleep disturbance, the timeframe of sleep disturbance, and the quality score of study evaluation, which is provided in the Appendix in S1 File.

The quality of the included articles was assessed using the 11-item methodological checklist recommended by the Agency for Healthcare Research and Quality (AHRQ) [11] for cross-sectional studies (https://www.ncbi.nlm.nih.gov/books/NBK35156/). An item would be scored "1" only if it was answered as “Yes”; the item would be scored “0” when it was answered “No” or “Unclear”. The quality of the study was assessed as (1) “low” when the score was 0–3; (2) “moderate” when the score was 4–7; and (3) “high” when the score was 8–11. Studies classified as “low” quality were excluded from further meta-analysis. Any discrepancy in assessment was also resolved by the third reviewer when necessary.

### Data analysis

We used STATA version 14.0 (Stata Corporation, College Station, TX, USA) and the fixed/random-effects models to calculate pooled estimates for sleep disturbance prevalence and corresponding 95% confidence intervals (CIs). Between-study heterogeneity was tested with *I*^2^ statistic and Cochrane Q statistics, while random-effects models were used when *I*^2^>50% and *p*<0.10 indicated significant heterogeneity and *I*^2^>75% indicated high heterogeneity [12, 13]. Subgroup analyses were performed to compare the differences in gender, area, assessment tools, effective sample size, and study stage with the chi-square test. Furthermore, the meta-regression analysis, using the Knapp-Hartung modification method, was performed to explore the influence of moderators on heterogeneity. Publication bias was conducted using Begg and Egger tests and funnel plots [14, 15]. Sensitivity analyses were conducted to test the consistency and quality of pooled results by removing each study individually. All tests were two-tailed with a statistically significant threshold of *P* <0.05.

## Results

### Studies retrieved and description

**Fig 1:**
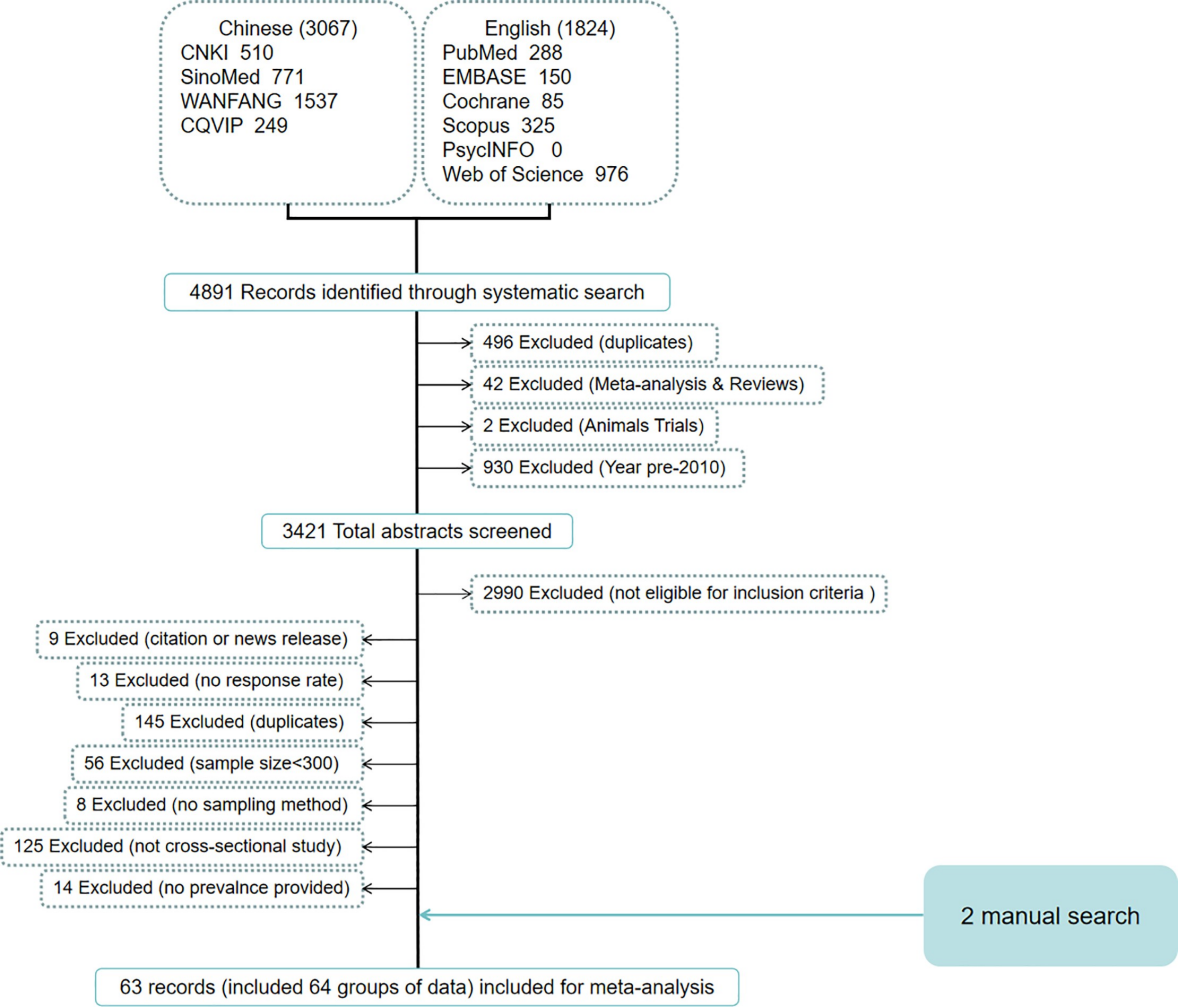
Screening process.

Our search strategy identified 4,891 potentially eligible records. We screened the total abstracts of 3,421 records after removing duplicates, reviews, animal experiments, and articles published before 2010, of which 431 full-texts were read, and 61 met the inclusion criteria. Hand searching contributed an additional two articles, resulting in a total of 63 articles (64 groups of outcomes) for the analyses (Fig 1).

A total of 430,422 subjects (males: 50.08%) were assessed, covering 28 mainland cities and 14 administrative provinces/municipalities, including Beijing, Shanghai, Hong Kong, Macau, and Taiwan. A total of 53 studies reported their prevalence of sleep disturbances using the Pittsburgh Sleep Quality Index (PSQI) [16], while five studies used the Insomnia Severity Index (ISI) [17], and three studies used three subtypes of insomnia [difficulty initiating sleep (DIS), difficulty maintaining sleep (DMS), and early morning awakening (EMA)], based on the International Classification of Sleep Disorders Diagnoses, Second Edition (ICSD-2) [18] from the American Academy of Sleep Medicine (2005). One study defined sleep disturbance using the Insomnia Self-Assessment Inventory (ISAI) from Social Development Trend Survey [19, 20] in three scales: difficulty in initiating sleep (DIS), difficulty in maintaining sleep (DMS), and nonrestorative sleep (NRS). The participants from 11 survey studies were from junior high school, while 24 studies focused on students from senior high school. The other studies had students from both schools, see details in Table 1.

**Table 1:** Quality assessment of cross-sectional studies with AHRQ methodological checklist.

Author	1	2	3	4	5	6	7	8	9	10	11	Total Score
Ge and Li^**2010**^	Yes	Yes	Yes	No	Not Clear	No	Yes	No	No	Yes	No	5
Liu^**2010**^	Yes	Yes	Yes	No	Not Clear	Yes	Yes	No	No	Yes	No	6
Ye^**2010**^	Yes	Yes	Yes	No	Not Clear	Yes	Yes	No	No	Yes	No	6
Liu^**2010**^	Yes	Yes	Yes	No	Not Clear	Yes	Yes	Yes	No	Yes	No	7
Liu, et al^**2010**^	Yes	Yes	Yes	No	Not Clear	No	Yes	No	No	Yes	No	5
Mak, et al^**2010**^	Yes	Yes	Yes	No	Not Clear	Yes	Yes	No	Yes	Yes	No	7
Liu, et al^**2011**^	Yes	Yes	No	No	Not Clear	No	Yes	No	No	Yes	No	4
Liu, et al^**2011**^	Yes	Yes	Yes	No	Not Clear	No	Yes	No	Yes	Yes	No	6
Liu, et al^**2011**^	Yes	Yes	Yes	No	Not Clear	No	Yes	No	No	Yes	No	5
Zeng^**2011**^	Yes	Yes	No	No	Not Clear	No	Yes	No	No	Yes	No	4
Cheung and Wong^**2011**^	Yes	Yes	No	No	Not Clear	No	Yes	No	Yes	Yes	No	5
Zhang, et al^**2012**^	Yes	Yes	Yes	No	Not Clear	No	Yes	No	No	Yes	No	5
Luo, et al^**2012**^	Yes	Yes	No	No	Not Clear	No	Yes	No	Yes	Yes	No	5
Liu, et al^**2012**^	Yes	Yes	Yes	No	Not Clear	No	No	No	No	Yes	No	4
Han^**2012**^	Yes	Yes	Yes	No	Not Clear	No	No	No	No	Yes	No	4
Zhou, et al^**2012**^	Yes	Yes	Yes	No	Not Clear	Yes	Yes	Yes	No	Yes	No	7
Cheng, et al^**2012**^	Yes	Yes	Yes	No	Not Clear	No	Yes	No	No	Yes	No	5
Pan, et al^**2012**^	Yes	Yes	Yes	No	Not Clear	No	Yes	Yes	No	Yes	No	6
Xu, et al^**2012**^	Yes	Yes	No	No	Not Clear	Yes	Yes	Yes	No	Yes	No	6
Wang^**2013**^	Yes	Yes	No	No	Not Clear	Yes	Yes	No	Yes	Yes	No	6
Ren, et al^**2013**^	Yes	Yes	No	No	Not Clear	Yes	Yes	No	No	Yes	No	5
Du, et al^**2013**^	Yes	Yes	Yes	No	Not Clear	No	Yes	No	No	Yes	No	5
Yan, et al^**2013**^	Yes	Yes	Yes	No	Not Clear	No	Yes	No	No	Yes	No	5
Peng, et al^**2013**^	Yes	Yes	Yes	No	Not Clear	Yes	Yes	No	Yes	Yes	No	7
Chen, et al^**2013**^	Yes	Yes	Yes	No	Not Clear	Yes	Yes	No	No	Yes	No	6
Zhang, et al^**2013**^	Yes	Yes	No	No	Not Clear	Yes	Yes	No	No	Yes	No	5
Wang, et al^**2014**^	Yes	Yes	No	No	Not Clear	No	Yes	No	No	Yes	No	4
Ning, et al^**2014**^	Yes	Yes	Yes	No	Not Clear	No	Yes	No	No	Yes	No	5
Xu, et al^**2014**^	Yes	Yes	Yes	No	Not Clear	No	Yes	No	No	Yes	No	5
Cui^**2014**^	Yes	Yes	No	No	Not Clear	Yes	Yes	No	No	Yes	No	5
Yang, et al^**2014**^	Yes	Yes	No	No	Not Clear	No	Yes	No	No	Yes	No	4
Chen, et al^**2014**^	Yes	Yes	Yes	No	Not Clear	Yes	Yes	No	Yes	Yes	No	7
Guo, et al^**2014**^	Yes	Yes	No	No	Not Clear	Yes	Yes	No	No	Yes	No	5
Zhu, et al^**2015**^	Yes	Yes	No	No	Not Clear	No	Yes	No	No	Yes	No	4
Zhou & Yao^**2015**^	Yes	Yes	Yes	No	Not Clear	Yes	Yes	Yes	No	Yes	No	7
Jing, et al^**2015**^	Yes	Yes	Yes	No	Not Clear	No	Yes	No	No	Yes	No	5
Hou, et al^**2015**^	Yes	Yes	Yes	No	Not Clear	No	Yes	No	No	Yes	No	5
Huang, et al^**2015**^	Yes	Yes	No	No	Not Clear	No	Yes	No	No	Yes	No	4
Bao, et al^**2016**^	Yes	Yes	No	No	Not Clear	Yes	Yes	No	No	Yes	No	4
Tan, et al^**2016**^	Yes	Yes	Yes	No	Not Clear	Yes	Yes	No	No	Yes	No	6
Zhang, et al^**2016**^	Yes	Yes	Yes	No	Not Clear	Yes	Yes	No	No	Yes	No	6
Zhang, et al^**2016**^	Yes	Yes	No	No	Not Clear	Yes	Yes	No	No	Yes	No	5
Tao^**2016**^	Yes	Yes	No	No	Not Clear	No	Yes	No	No	Yes	No	4
Li, et al^**2017**^	Yes	Yes	Yes	No	Not Clear	Yes	Yes	Yes	No	Yes	No	7
Yang, et al^**2017**^	Yes	Yes	No	No	Not Clear	No	Yes	No	No	Yes	No	4
Du, et al^**2017**^	Yes	Yes	No	No	Not Clear	No	Yes	No	No	Yes	No	4
Luo, et al^**2017**^	Yes	Yes	Yes	No	Not Clear	Yes	Yes	No	No	Yes	No	6
Li, et al^**2017**^	Yes	Yes	Yes	No	Not Clear	Yes	Yes	No	No	Yes	No	6
Zhang, et al^**2017**^	Yes	Yes	Yes	No	Not Clear	No	Yes	No	No	Yes	No	5
Yao, et al^**2017**^	Yes	Yes	Yes	No	Not Clear	Yes	Yes	Yes	No	Yes	No	7
Zhou, et al^**2017**^	Yes	Yes	Yes	No	Not Clear	No	Yes	No	No	Yes	No	5
Chen, et al^**2017**^	Yes	Yes	Yes	No	Not Clear	No	Yes	No	No	Yes	No	5
Shen^**2018**^	Yes	Yes	No	No	Not Clear	Yes	Yes	Yes	No	Yes	No	6
Wang^**2018**^	Yes	Yes	Yes	No	Not Clear	Yes	Yes	No	No	Yes	No	6
Huang, et al^**2018**^	Yes	Yes	Yes	No	Not Clear	Yes	Yes	No	No	Yes	No	6
Wu^**2018**^	Yes	Yes	Yes	No	Not Clear	Yes	Yes	No	No	Yes	No	6
Fan, et al^**2018**^	Yes	Yes	Yes	No	Not Clear	Yes	Yes	No	No	Yes	No	6
Li^**2019**^	Yes	Yes	No	No	Not Clear	Yes	Yes	No	No	Yes	No	5
Liu^**2019**^	Yes	Yes	Yes	No	Not Clear	Yes	Yes	Yes	No	Yes	No	7
Wu, et al^**2019**^	Yes	Yes	Yes	No	Not Clear	Yes	Yes	No	No	Yes	No	6
Xiao, et al^**2020**^	Yes	Yes	Yes	No	Not Clear	Yes	Yes	No	No	Yes	No	6
Chan, et al^**2020**^	Yes	Yes	Yes	No	Not Clear	No	Yes	No	No	Yes	No	5
Luo^**2020**^	Yes	Yes	No	No	Not Clear	No	Yes	No	No	Yes	No	4

*Article quality was assessed as follows: low quality = 0–3; moderate quality = 4–7; high quality = 8–11.

1. Define the source of information (survey, record review).

2. List inclusion and exclusion criteria for exposed and unexposed subjects (cases and controls) or refer to previous publications.

3. Indicate time period used for identifying patients.

4. Indicate whether or not subjects were consecutive if not population-based.

5. Indicate if evaluators of subjective components of study were masked to other aspects of the status of the participants.

6. Describe any assessments undertaken for quality assurance purposes (e.g., test/retest of primary outcome measurements).

7. Explain any patient exclusions from analysis.

8. Describe how confounding was assessed and/or controlled.

9. If applicable, explain how missing data were handled in the analysis.

10. Summarize patient response rates and completeness of data collection.

11. Clarify what follow-up, if any, was expected and the percentage of patients for which incomplete data or follow-up was obtained.

### Methodological quality of studies

The quality of the included studies, as assessed by AHRQ, is shown in Table 2. The median study quality score was 5 (range 4–7). A total of 22 studies (34.92%) failed to indicate the exact time of the survey. Thirteen studies (20.63%) were scored 4, nine studies (14.29%) were scored 7, and the remaining were scored 5 and 6. None of the studies were scored less than 4 with low quality.

**Table 2:** Characteristics of studies included in this meta-analysis.

Study	Survey Time	Area	Sampling Method	Effective Sample	Response Rate (%)	Proportion of Males (%)	Age (Mean ± SD)	Studying Stage	Assessment Instrument	Cut-off	Prevalence of Sleep disturbance (%)	Time Frame	Quality Score
Ge and Li^2010^ [21]	2010	Hangzhou-S	C	341	96.88	60.80	12–18	J	PSQI	≥8	21.99	LM	5
Liu^2010^ [22]	2010	Fuzhou-S	R	757	86.20	47.56	15.26±2.27	J, S	PSQI	≥8	15.85	LM	6
Ye^2010^ [23]	2009	Macau-S	R, C	501	93.64	51.50	15.38±1.328	J	PSQI	≥8	25.20	LM	6
Liu^2010^ [5]	2010	Taiyuan-N	R	439	97.50	47.20	13–16	J	PSQI	≥8	27.40	LM	7
Liu, et al^2010^ [24]	2010	Fuzhou-S	S	381	84.70	51.44	16–20	S	PSQI	≥8	31.50	LM	5
Mak, et al^2010^ [25]	2010	Hong Kong-S	S, R	28839	84.80	49.70	12–18	J, S	DIS/DMS/EMA ICSD-2	≥3 times/week	35.10	LM	7
Liu, et a^l2011^ [26]	NR	Fuzhou-S	S, R	566	94.30	44.88	NR	J, S	PSQI	≥8	17.10	LM	4
Liu, et al^2011^ [27]	2009	Nanchang-S	S, C	950	95.00	49.79	14.9±0.76	J	PSQI	≥8	17.68	LM	6
Liu, et al^2011^ [28]	2010	Fuzhou-S	S, R	609	93.69	47.13	12–19	J, S	PSQI	≥8	15.90	LM	5
Zeng^2011^ [29]	NR	Zhejiang Province-S	S, R	354	97.00	49.20	16.5±1.2	S	PSQI	≥8	32.80	LM	4
Cheung & Wong^2011^ [30]	2010	Hong Kong-S	R	719	100.00	60.40	10–14	J, S	PSQI	≥5\6	30.70	LM	5
Zhang, et al^2012^ [31]	2010	Xuzhou-S	S, C	1385	99.00	54.00	NR	J, S	PSQI	≥8	36.60	LM	5
Luo, et al^2012^ [32]	NR	Guangdong Province-S	C, R	4800	96.00	48.10	14.9±1.36	J, S	ISI	≥8	38.20	LTW	5
Liu, et al^2012^ [33]	2009	Guangdong Province-S	C	344	100.00	45.10	14–20	S	PSQI	≥8	37.80	LM	4
Han^2012^ [34]	2010	Beijing-N	S, C	552	95.00	52.50	NR	E, J, S	PSQI	≥8	12.86	LM	4
Zhou, et al^2012^ [35]	2008	Shanghai-S	R	1221	99.00	46.60	12–18	J, S	PSQI	≥5	34.32	LM	7
Cheng, et al^2012^ [36]	2008	Taiwan-S	R	2360	83.50	66.00	NR	NR	PSQI	≥6	54.70	LM	5
Pan, et al^2012^ [37]	2009	Heshan-S	J, R	861	95.70	47.60	15.3±1.8	S	DIS/DMS/EMA ICSD-2	≥3 times/week	22.90	LM	6
	2009	Macau-S	J, R	618	95.10	41.90	15.6±1.8	S	DIS/DMS/EMA ICSD-2	≥3 times/week	16.50	LM	6
Xu, et al^2012^ [38]	NR	Hefei-S	R	5226	97.90	55.70	17.36±2.93	J, S, U	PSQI	≥8	20.00	LM	6
Wang^2013^ [39]	NR	Hefei-S	C	542	90.00	0.90	18.69±1.69	S	PSQI	≥8	26.80	LM	6
Ren, et al^2013^ [40]	NR	Weifang-N	C, R	852	100.00	49.06	13.65±1.01	J	PSQI	≥7	10.10	LM	5
Du, et al^2013^ [41]	2011	Gaomi-N	R, C	1063	97.40	48.60	15.21±0.73	J	PSQI	≥8	8.00	LM	5
Yan, et al^2013^ [42]	2011	Changsha-S	S, R	2216	96.94	51.80	14.6±1.56	J, S	PSQI	≥8	14.40	LM	5
Peng, et al^2013^ [43]	2012	Urmqi-N	C	1096	99.27	49.73	16.2±0.74	S	PSQI	≥8	21.10	LM	7
Chen, et al^2013^ [44]	2001	Taiwan-S	S	2113	100.00	52.86	15–17	S	DIS/DMS/NRS-ISAI	≥3 times/week	20.90	LM	6
Zhang, et al^2013^ [45]	NR	Xuzhou-S	R	1052	87.70	52.76	NR	J	PSQI	≥8	20.20	LM	5
Wang, et al^2014^ [46]	NR	Shandong Province-N	R, C	1227	81.80	44.40	NR	S	PSQI	≥8	27.20	LM	4
Ning, et al^2014^ [47]	2012–2013	Xuzhou-S	C, R	4729	100.00	52.46	NR	J, S	PSQI	≥8	26.40	LM	5
Xu, et al^2014^ [48]	2009	Shanghai-S	C	301	99.30	40.53	16–17	S	PSQI	≥8	11.63	LM	5
Cui^2014^ [49]	NR	He’nan Province-N	S, R	1406	100.00	43.17	NR	S	PSQI	≥8	29.20	LM	5
Yang, et al^2014^ [50]	NR	Chengdu-S	C	1180	90.77	52.20	16.58±0.57	S	PSQI	≥8	28.47	LM	4
Chen, et al^2014^ [51]	2011	Shenyang/Xinxiang/Chongqing/Guangzhou-NS	C	13817	99.00	48.10	14.8±1.8	J, S	PSQI	≥8	26.50	LM	7
Guo, et al^2014^ [52]	NR	Guangdong Province-S	S, R, C	3186	95.20	53.40	15.6±1.6	J, S	PSQI	≥8	39.60	LM	5
Zhu, et al^2015^ [53]	NR	Liu’an-S	C	543	90.00	56.70	18.62	S	PSQI	≥8	28.00	LM	4
Zhou & Yao^2015^ [54]	2012	Shanghai-S	R	1173	91.36	50.13	12.11–17.71	J, S	PSQI	≥8	18.84	LM	7
Jing, et al^2015^ [55]	2015	Ningbo-S	J, C	3932	99.70	51.60	15.08±1.54	J, S	ISI	≥8	25.30	LTW	5
Hou, et al^2015^ [56]	2014	Shen Zhen-N	S, R, C	1175	100.00	52.51	NR	S	PSQI	≥8	34.40	LM	5
Huang, et al^2015^ [57]	NR	Nanchang-N	S, C	608	95.30	51.81	14–18	S	PSQI	≥8	20.20	LM	4
Bao, et al^2016^ [58]	NR	Guangdong Province-S	R, C	1053	100.00	45.20	14.95±1.66	S	PSQI	≥8	15.60	LM	4
Tan, et al^2016^ [59]	2012	Shantou-S	R	1661	96.20	51.80	14.53	S	PSQI	≥5	40.00	LM	6
Zhang, et al^2016^ [60]	2011–2012	Hong Kong-S	R	7507	55.20	51.50	8–17	E, J, S	DIS/DMS/EMA ICSD-2	≥3 times/week	25.79	LM	6
Zhang, et al^2016^ [61]	NR	Yinchuan-N	S, C	3360	98.82	50.90	17.62±1.03	S	PSQI	≥8	27.53	LM	5
Tao^2011^ [62]	NR	Guangyuan-S	R	386	96.50	52.07	18.5±1.6	S	PSQI	≥8	23.32	LM	4
Li, et al^2017^ [63]	2014–2015	Guangdong/Liaoning/Shandong/Hu’nan/Shanxi/ Guizhou-NS	S, C	123459	95.93	52.20	15.04	J, S	PSQI	≥8	22.41	LM	7
Yang, et al^2017^ [64]	NR	Zhongshan-S	C	1323	98.66	60.24	NR	J	PSQI	≥8	27.51	LM	4
Du, et al^2017^ [65]	NR	He’nan/Hu’nan Province-NS	C	305	97.00	40.00	11–17	J, S	PSQI	≥8	19.34	LM	4
Luo, et al^2017^ [66]	2014	Guangzhou-S	R	3342	70.61	80.00	17.67±1.73	S	ISI	>8	29.83	LTW	6
Li, et al^2017^ [4]	2015	Shenzhen-S	S, C	860	95.56	50.35	14.34±1.27	J	PSQI	≥8	16.51	LM	6
Zhang, et al^2017^ [67]	2015–2016	Shenzhen-S	S, C	3168	98.54	52.50	15.01±1.76	J, S	PSQI	≥8	22.00	LM	5
Yao, et al^2017^ [68]	2016	Shenyang-N	C	775	95.10	53.16	15.58±1.65	J, S	PSQI	≥8	18.30	LM	7
Zhou, et al^2017^ [3]	2016	Xuzhou-S	R	600	100.00	51.67	NR	NR	ISI	>8	32.00	LTW	5
Chen, et al^2017^ [69]	2006–2007	Nanyang-N	S, C	360	97.63	50.56	16.30	S	PSQI	≥8	33.61	LM	5
Shen^2018^ [70]	NR	Ganzhou-S	S, R	3081	97.01	50.80	NR	J	PSQI	≥8	10.30	LM	6
Huang^2018^ [71]	2014	Nanchang-S	S, C, R	608	95.30	51.81	16.27±0.87	S	PSQI	≥8	20.20	LM	6
Huang, et al^2018^ [72]	2016	Guangzhou-S	S, C	5781	91.76	44.56	12–20	J, S	PSQI	≥8	33.10	LM	6
Wu^2018^ [7]	2016	Urmqi-N	R	8242	93.73	48.80	14.54±1.79	J, S	PSQI	≥8	28.44	LM	6
Fan, et al^2018^ [8]	2016	Yinchuan-N	C	2116	83.93	42.16	17.17±0.94	S	PSQI	≥8	38.04	LM	6
Li^2019^ [73]	NR	Yancheng-N	J	625	95.00	57.40	NR	J	PSQI	≥8	33.00	LM	5
Liu^2019^ [6]	2017–2018	Shanghai-S	S, C, R	1986	85.00	51.30	14.71±2.01	J, S	ISI	>8	37.00	LTW	7
Wu, et al^2019^ [74]	2014–2015	Shanxi Province-N	S, C	9560	100.00	44.80	15.3±1.8	J, S	PSQI	≥8	23.60	LM	6
Xiao, et al^2019^ [75]	2015	Guangdong/Liaoning/Shandong/Hunan/Shanxi/Chongqing/Guizhou-NR	S, C, R	153547	96.18	48.00	15±1.8	J, S	PSQI	≥8	21.60	LM	6
Chan, et al^2020^ [76]	2017–2019	Hong Kong-S	R	1667	61.10	56.50	14.8±1.6	J, S	DIS/DMS/EMA ICSD-2	≥3 times/week	37.00	LM	5
Luo^2020^ [77]	NR	Huizhou-S	C	1016	98.00	45.00	15~16	S	PSQI	≥8	47.00	LM	4

Area: S-Southern China, N-Northern China. Sampling Method: C-Cluster sampling; R-Random sampling; S-Stratified sampling; J-Judgmental sampling. Studying Stage: E-Elementary School, J-Junior High School, S-Senior High School. Timeframe: LM-Last Month, LTW-Last Two Weeks. PSQI: Pittsburgh Sleep Quality Index; ISA: Insomnia Severity Index; ICSD-2: International Classification of Sleep Disorders diagnoses, Second Edition; ISAI: Insomnia Self-Assessment Inventory.

### Prevalence of sleep disturbances

**Fig 2:**
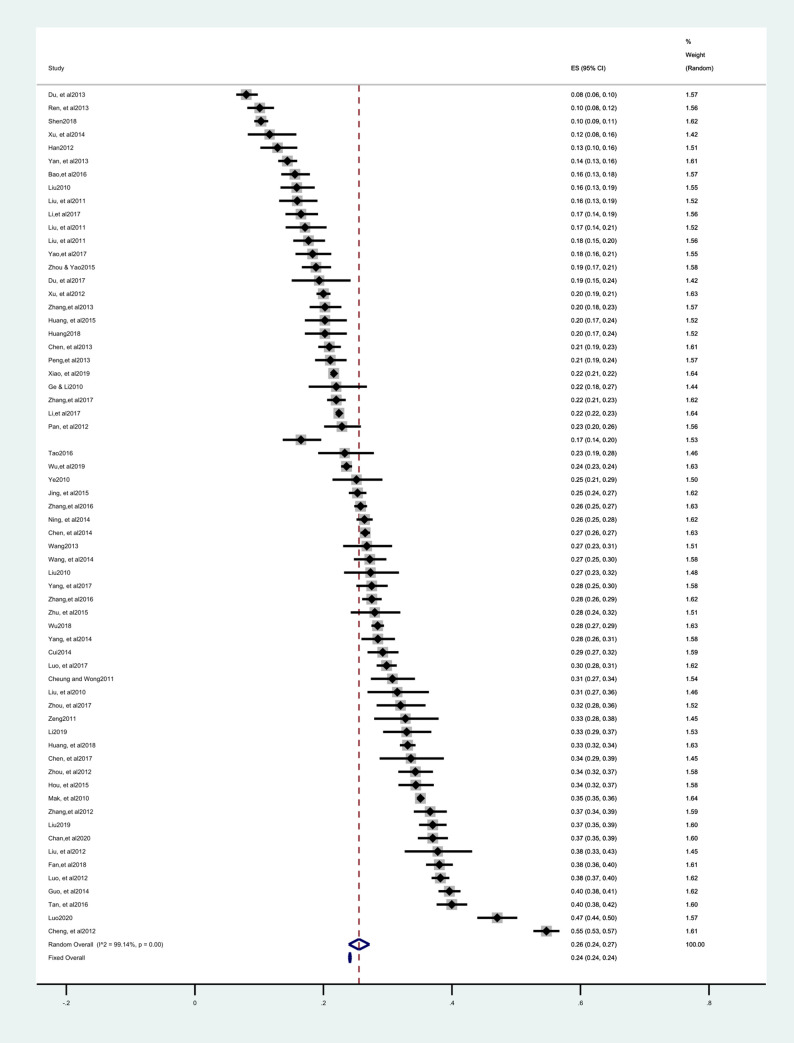
Forest plot of pooled estimates for prevalence.

In total, the 63 eligible studies included 64 groups of outcomes (one study had two groups). For the meta-analysis, the prevalence of sleep disturbances in adolescence ranged from 8% in Gaomi to 54.7% in Taiwan, while the pooled prevalence was 26% (95% CI: 24–27%). Substantial heterogeneity existed between the studies, *I*^2^ = 99.14%, chi-square = 1871.42, *p* <0.001, τ^2^ = 0.02 (Fig 2). There were no significant changes in the results after removing each study sequentially.

### Subgroup analyses

The estimates of sleep disturbance prevalence from the subgroup analysis, determined for gender, area, sample size, studying stage, survey time, instruments, PSQI cut-off, and quality score of study, are shown in Table 3. Compared to other sample sizes, effective samples of more than 1,000 and less than 3,000, indicated the highest pooled prevalence of 30% (95% CI: 24–35%, *p*<0.001). The prevalence of sleep disturbance in senior high school students (28%, 95% CI: 24–31%, *p*<0.001) was higher than junior high school students (20%, 95% CI: 15–24%, *p*<0.001). The subgroup of the sample size and studying stage indicated statistically significant heterogeneity with Q11.99 (*p* = 0.01) and Q7.77 (*p* = 0.01). There were no statistically significant differences in heterogeneity between the other groups.

**Table 3:** Subgroup analyses of studies included in this meta-analysis.

Subgroup Analysis	Events	Sample Size	Prevalence (%)	95% CI (%)	Weight (%)	I^2	p	Q(p) Heterogeneity between-group	Begg’s test z (p)	Egger’s test t (p)
**Gender (22)**										
Male (22)	10825	38189	28	24–33	49.96	98.97	p<0.001	0.12 (0.72)	0.652 (0.419)	0.16 (0.872)
Female (22)	10123	35923	27	24–31	50.04	98.16	p<0.001		0.612 (0.653)	-0.30 (0.770)
**Area (59)**										
North China (16)	8664	33456	24	21–28	26.69	98.56	p<0.001	0.89 (0.34)	0.05 (0.964)	-0.04 (0.967)
South China (43)	31585	105838	27	24–30	73.31	99.1	p<0.001		0.39 (0.696)	-0.97 (0.338)
**Sample Size (63)**										
<500 (10)	992	3712	26	21–31	14.49	92.19	p<0.001	11.99 (0.01*)	2.15 (0.032)	4.10 (0.003)
500–1000 (16)	2384	11645	21	18–24	26.23	94.8	p<0.001		3.38 (0.001)	6.48 (0.000)
1000–3000 (20)	9040	29489	30	24–35	31.65	99.61	p<0.001		1.27 (0.206)	2.68 (0.015)
>3000 (17)	92387	385576	27	24–29	27.63	99.14	p<0.001		1.19 (0.232)	2.01 (0.063)
**Studying Stage (34)**										
Junior High School Students (11)	1902	11087	20	15–24	31.53	97.81	p<0.001	7.77 (0.01*)	1.87 (0.062)	4.07 (0.003)
Senior High School Students (23)	7645	26651	28	24–31	68.47	97.15	p<0.001		0.60 (0.552)	0.12 (0.904)
**Survey Time (41)**										
2010–2015 (28)	24153	81319	25	22–29	68.3	99.11	p<0.001	0.83 (0.36)	0.39 (0.694)	-1.68 (0.104)
2015–2019 (13)	71672	315693	27	25–29	31.7	98.94	p<0.001		0.43 (0.669)	2.98 (0.013)
**Instruments (63)**										
PSQI (53)	86634	374157	25	24–27	82.49	98.87	p<0.001	3.41 (0.06)	0.49 (0.623)	1.81 (0.076)
Others (10)	18169	56265	29	25–33	17.51	98.74	p<0.001		0.31 (0.755)	-1.42 (0.190)
**PSQI Cut-Off (53)**										
≥8 (48)	83953	367344	24	23–26	90.45	98.51	p<0.001	1.27 (0.26)	0.48 (0.631)	1.36 (0.182)
Others (5)	2681	6813	34	17–51	9.55	99.59	p<0.001		0.73 (0.462)	-0.03 (0.978)
**Quality Score (63)**										
4 (13)	2514	9457	26	21–31	19.71	97.22	p<0.001	2.50 (0.47)	1.16 (0.246)	0.89 (0.392)
5 (23)	11961	40287	28	23–32	35.88	99.18	p<0.001		0.26 (0.792)	0.38 (0.705)
6 (18)	47009	207873	24	21–26	30.16	98.92	p<0.001		0.07 (0.944)	1.04 (0.314)
7 (9)	43319	172805	27	22–32	14.26	99.60	p<0.001		0.10 (0.917)	0.88 (0.406)

*p<0.05

### Meta-regression analyses

The meta-regression was performed using the Knapp-Hartung modification method. Further details are presented in Table 4. The sample size between 1,000 and 3,000 (τ^2^ = 0.006994, *p* = 0.004<0.05) and studying stage (τ^2^ = 0.005651, *p* = 0.009<0.05) were statistically significant, which accounted for 9.78% and 20.83% of between-study heterogeneity, respectively. There were no statistically significant contributions to heterogeneity in mean age, sample size, survey time, the quality score of study (all *p*-values >0.05). No model was created that included all factors to explain the heterogeneity of this meta-analysis.

**Table 4:** Meta-regression analysis.

Categories	Exp (B)	Standard Error	Z-value	P-value	[95% CI]		Tau^2^	Adjusted R^2^ (%)
**Mean Age (37)**	0.009947	0.122517	0.81	0.422	0.9852099	1.035407	0.007496	0
**Sex Ratio (F/M) (63)**	1.000031	0.00091	0.03	0.973	0.9982132	1.001851	0.007877	0
**Response Rate (63)**	0.9976231	0.0013064	-1.82	0.074	0.9950149	1.000238	0.007435	4.09
**Sample Size (63)** n = 1000–3000	1.092256 (Coef.)	0.0325041	2.97	0.004*	1.029135	1.159248	0.006994	9.78
**Studying Stage (35)**	0.0842858 (Coef.)	0.0304005	2.77	0.009*	0.0224354	0.1461361	0.005651	20.83%
**Survey Time (41)**	1.002648	0.0044096	0.6	0.551	0.9937753	1.0116	0.008077	0
**Quality Score (63)**	0.995359	0.0122886	-0.38	0.708	0.9710951	1.020229	0.007868	0

*p<0.05

### Publication bias

**Fig 3:**
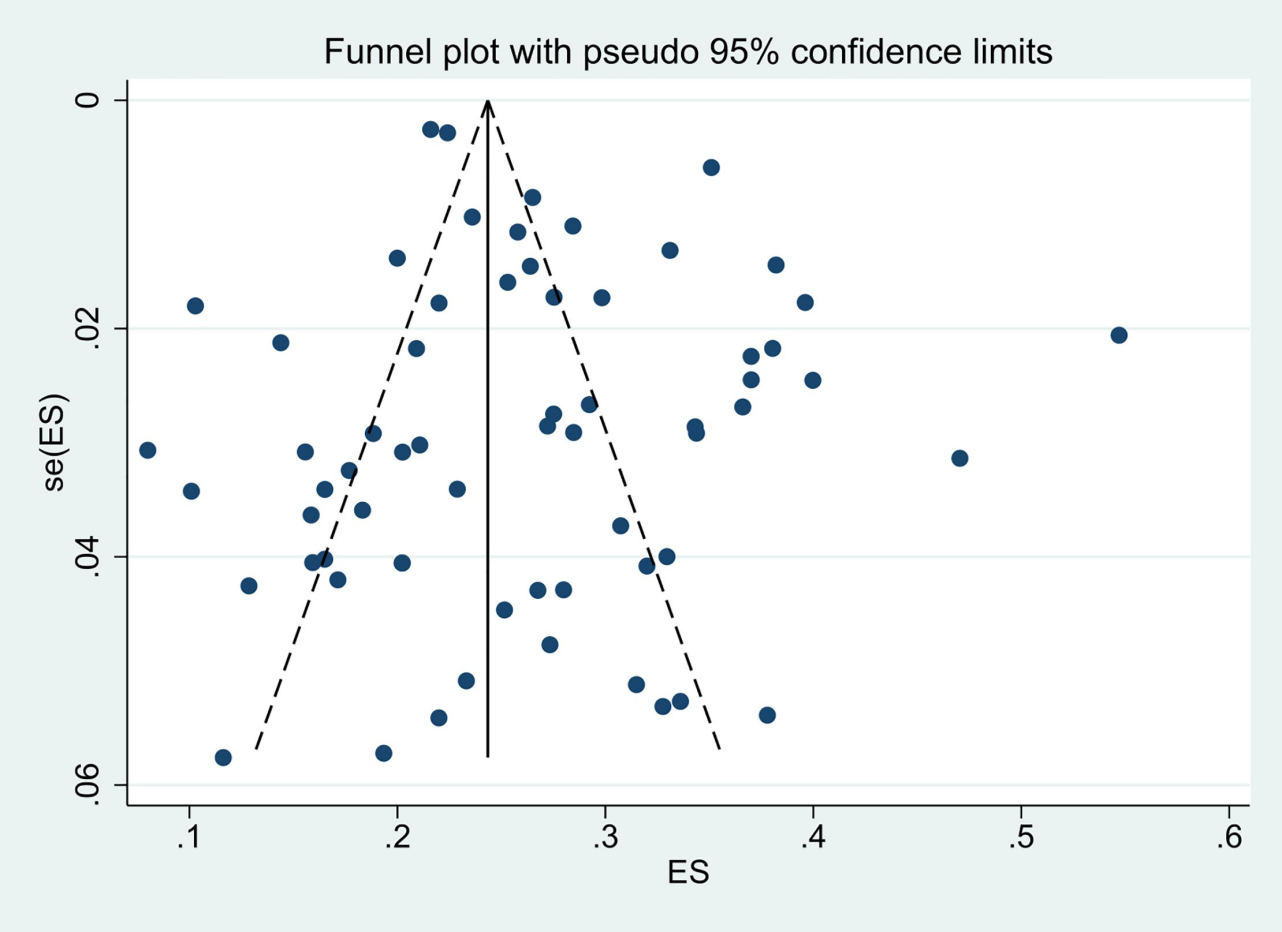
Funnel plot of publication bias.

Publication bias was found using visual inspection of the funnel plot and Egger’s test (t = 2.04, *p =* 0.046), as shown in Fig 3. The trim-and-fill method was used to eliminate bias by trimming nine studies, including Liu 2019, Chan *et al*. 2020, Liu *et al*. 2012, Fan *et al*. 2018, Luo *et al*. 2012, Guo *et al*. 2014, Tan *et al*. 2016, Luo 2020, and Cheng *et al*. 2012 [32, 33, 36, 52, 59, 71, 73, 76, 77]. When this was done, the estimated prevalence was reduced to 23.6%.

## Discussion

This meta-analysis, which pooled 430,422 adolescents in junior/senior high schools, proved the existence of sleep disturbances with an average prevalence of 26% (95% CI: 24–27%). This rate is higher than the pooled prevalence of university students at 25.7% (95% CI: 22.5–28.9%) and adults (<43.7 years old) at 20.4% (95% CI: 14.2–28.2%) [78, 79]. In addition, this was even higher than the pooled prevalence of insomnia in the general Chinese population at 15% (95% CI: 12.1–18.5%) [79]. It is possible that a trend may exist, in which the prevalence of sleep disturbance is lower during junior high school (20%, 95% CI: 15–24%), followed by an increase in senior high school (28%, 95% CI: 24–31%), a reduction in adulthood [79], followed by an increase to the highest pooled prevalence level of 35.9% in older Chinese adults (95% CI: 30.6–41.2%) [80]. The pooled prevalence of sleep disturbances reached the first peak in older adolescents.

Age is commonly associated with the degree of disturbance in Chinese adolescents [35, 38, 81]; however, our findings show that the prevalence increases with the studying stage, rather than the mean age. Senior high school students had a higher pooled prevalence of sleep disturbance compared to junior high school students (28%, 95% CI: 24–31% vs. 20%, 95% CI: 15–24%) in China, which may be attributed to academic pressure, insufficient sport duration, and increased anxiety/depression levels [33, 34, 56]. The difference in the studying stage accounted for 20.83% of the variation for between-study heterogeneity (τ^2^ = 0.005651, *p =* 0.009).

Gender did not affect the pooled prevalence in our results, which is inconsistent with other studies [31, 36, 52, 55, 66]. There were also no differences in the sex ratio, geographical location, survey time, or quality evaluation of the studies. In total, 84.13% of studies used PSQI and 79.19% used PSQI, with a cut-off ≥8, and there were no significant differences between PSQI and other instruments and between PSQI cut-off ≥8 and others through the sub-group analyses. This was likely due to representation issues.

Statistically significant differences were also found in the sample size (<500 (26%, 95% CI [24–29%]) vs. 500–100 (21%, 95% CI [18–24%]) vs. 1,000–3,000 (30%, 95% CI [24–35%]) vs. >3000 (27%, 95% CI [24–29%]). Furthermore, the sample size of 1,000–3,000 contributed to 9.78% (τ^2^ = 0.006994, *p* = 0.004*) of the heterogeneity.

All of the included studies with “moderate” quality pooled 104,802 adolescents with sleep disturbances, covering 14 administrative provinces and 28 cities across China, and showed a good representation of Chinese adolescents. Approximately 79.37% of the included studies response rates were more than 90%. In this meta-analysis, we used sub-group analyses and meta-regression analyses to explore the sources of heterogeneity, but we could not find an overall explanation of the heterogeneity sources. Thus, several limitations should be addressed. First, some of the studies did not report the exact survey time, which is important for students. Sleep quality is different between school days and non-school days, and between exam periods and non-exam periods [82]. Second, most studies failed to report issues of study quality control that could affect the reliability and validity of the study results. Third, some detailed characteristics of the subjects were not presented. The meta-analysis was based on published data, which could lead to difficulties in further sub-group analyses and meta-regression analyses. In addition, we believe that sub-group analyses of different types of sleep disturbances in Chinese adolescents by severity degree should be taken into consideration in future research, which will be useful to get to the point., Fourth, we could not eliminate the high level of heterogeneity in the prevalence estimate due to many different epidemiological survey studies.

In conclusion, the pooled prevalence of sleep disturbances in Chinese adolescents was 26% (95% CI, 24–27%), higher than the pooled prevalence in the general population (15%, 95% CI: 12.1–18.5%) in China. The sub-group analyses and meta-regression analyses indicated that higher prevalence was associated with studying stage and effective sample size, especially for adolescents in senior high school (28%, 95% CI: 24–31%) and sample sizes between 1,000 and 3,000 (30%, 95% CI: 24–35%). Further research is needed to better understand the sleep disturbances of adolescents to develop effective psychological and behavioral interventions.

## Supporting information

S1 File Appendix. Search strategies & quality assessment & PRISMA 2009 checklist.
(PDF)
