# Supplementary material for: Prevalence of sleep disturbances in Chinese adolescents: A systematic review and meta-analysis
Source: PLoS One. 2021 Mar 4;16(3):e0247333. doi: 10.1371/journal.pone.0247333 (PMC7932116; doi:10.1371/journal.pone.0247333)
Supplement: S1 File — Search strategies & quality assessment & PRISMA 2009 checklist. (PDF) [file pone.0247333.s001.pdf]

Appendix 1 Search Strategies of PubMed, EMBASE, Cochrane, Web of Science, Scopus , PsycINFO & SinoMed(in Chinese)

PubMed

((((((((Chinese[Title/Abstract]) OR (China[Title/Abstract])) OR (Macau[Title/Abstract])) OR (Hongkong[Title/Abstract])) OR (Taiwan[Title/Abstract])) OR (Taiwanese[Title/Abstract])) AND (((((((((((adolescents[Title/Abstract]) OR (teens[Title/Abstract])) OR (teenagers[Title/Abstract])) OR (youths[Title/Abstract])) OR (students[Title/Abstract])) OR (elementary school students[Title/Abstract])) OR (high school students[Title/Abstract]))) AND ((((prevalence[Title/Abstract]) OR (epidemiology survey[Title/Abstract])) OR (cross-section study[Title/Abstract])) OR (rate[Title/Abstract]))) AND ((Disorder, Sleep Wake[Title/Abstract]) OR (Disorders, Sleep Wake[Title/Abstract])) OR (Sleep Wake Disorder[Title/Abstract])) OR (Wake Disorder, Sleep[Title/Abstract])) OR (Wake Disorders, Sleep[Title/Abstract])) OR (Subwakefullness Syndrome[Title/Abstract])) OR (Subwakefullness Syndromes[Title/Abstract])) OR (Syndrome, Subwakefullness[Title/Abstract])) OR (Syndromes, Subwakefullness[Title/Abstract])) OR (Sleep Disorders[Title/Abstract])) OR (Disorder, Sleep[Title/Abstract])) OR (Disorders, Sleep[Title/Abstract])) OR (Sleep Disorder[Title/Abstract])) OR (Sleep-Related Neurogenic Tachypnea[Title/Abstract])) OR (Neurogenic Tachypnea, Sleep-Related[Title/Abstract])) OR (Neurogenic Tachypneas, Sleep-Related[Title/Abstract])) OR (Sleep Related Neurogenic Tachypnea[Title/Abstract])) OR (Sleep-Related Neurogenic Tachypneas[Title/Abstract])) OR (Tachypnea, Sleep-Related Neurogenic[Title/Abstract])) OR (Tachypneas, Sleep-Related Neurogenic[Title/Abstract])) OR (Long Sleeper Syndrome[Title/Abstract])) OR (Long Sleeper Syndromes[Title/Abstract])) OR (Sleeper Syndrome, Long[Title/Abstract])) OR (Sleeper Syndromes, Long[Title/Abstract])) OR (Syndrome, Long Sleeper[Title/Abstract])) OR (Syndromes, Long Sleeper[Title/Abstract])) OR (Short Sleeper Syndrome[Title/Abstract])) OR (Short Sleeper Syndromes[Title/Abstract])) OR (Sleeper Syndrome, Short[Title/Abstract])) OR (Sleeper Syndromes, Short[Title/Abstract])) OR (Syndrome, Short Sleeper[Title/Abstract])) OR (Syndromes, Short Sleeper[Title/Abstract])) OR (Short Sleep Phenotype[Title/Abstract])) OR (Phenotype, Short Sleep[Title/Abstract])) OR (Phenotypes, Short Sleep[Title/Abstract])) OR (Short Sleep Phenotypes[Title/Abstract])) OR (Sleep Phenotypes, Short[Title/Abstract])) OR ("Sleep Wake Disorders"[Mesh])) OR (((((((((((((((Intrinsic Sleep Disorder[Title/Abstract]) OR (Sleep Disorder, Intrinsic[Title/Abstract])) OR (Intrinsic Sleep Disorders[Title/Abstract])) OR (Sleep State Misperception[Title/Abstract])) OR (Sleep State Misperceptions[Title/Abstract])) OR (Hypersomnia, Post-Traumatic[Title/Abstract])) OR (Hypersomnia, Post Traumatic[Title/Abstract])) OR (Hypersomnias, Post-Traumatic[Title/Abstract])) OR (Post-Traumatic Hypersomnia[Title/Abstract])) OR (Post-Traumatic Hypersomnias[Title/Abstract])) OR (Hypersomnia, Posttraumatic[Title/Abstract])) OR (Hypersomnias, Posttraumatic[Title/Abstract])) OR (Posttraumatic Hypersomnia[Title/Abstract])) OR (Posttraumatic Hypersomnias[Title/Abstract])) OR ("Sleep Disorders, Intrinsic"[Mesh]))) OR (((((((((((((((((((((((((((Disturbed Nyctohemeral Rhythm[Title/Abstract]) OR (Disturbed Nyctohemeral Rhythms[Title/Abstract])) OR (Nyctohemeral Rhythm, Disturbed[Title/Abstract])) OR (Nyctohemeral Rhythms, Disturbed[Title/Abstract])) OR (Circadian Rhythm Sleep Disorders[Title/Abstract])) OR (Sleep-Wake Schedule Disorders[Title/Abstract])) OR (Sleep Wake Schedule Disorders[Title/Abstract])) OR (Sleep-Wake Schedule Disorder[Title/Abstract])) OR

(Sleep-Wake Cycle Disorders[Title/Abstract])) OR (Sleep Wake Cycle Disorders[Title/Abstract])) OR (Sleep-Wake Cycle Disorder[Title/Abstract])) OR (Shift-Work Sleep Disorder[Title/Abstract])) OR (Shift Work Sleep Disorder[Title/Abstract])) OR (Shift-Work Sleep Disorders[Title/Abstract])) OR (Sleep Disorders, Shift-Work[Title/Abstract])) OR (Sleep Disorder, Shift-Work[Title/Abstract])) OR (Sleep Disorder, Shift Work[Title/Abstract])) OR (Non-24 Hour Sleep-Wake Disorder[Title/Abstract])) OR (Non 24 Hour Sleep Wake Disorder[Title/Abstract])) OR (Sleep-Wake Disorder, Non-24 Hour[Title/Abstract])) OR (Sleep Wake Disorder, Non 24 Hour[Title/Abstract])) OR (Nonorganic Sleep Wake Cycle Disorders[Title/Abstract])) OR (Advanced Sleep Phase Syndrome[Title/Abstract])) OR (Delayed Sleep Phase Syndrome[Title/Abstract])) OR (Delayed Sleep-Phase Syndrome[Title/Abstract])) OR (Delayed Sleep-Phase Syndromes[Title/Abstract])) OR ("Sleep Disorders, Circadian Rhythm"[Mesh]))) OR (((((((((((((((((((((((((((Dyssomnia[Title/Abstract]) OR (Sleep Disorders, Extrinsic[Title/Abstract])) OR (Extrinsic Sleep Disorder[Title/Abstract])) OR (Extrinsic Sleep Disorders[Title/Abstract])) OR (Sleep Disorder, Extrinsic[Title/Abstract])) OR (Limit-Setting Sleep Disorder[Title/Abstract])) OR (Limit Setting Sleep Disorder[Title/Abstract])) OR (Limit-Setting Sleep Disorders[Title/Abstract])) OR (Sleep Disorders, Limit-Setting[Title/Abstract])) OR (Sleep Disorder, Limit-Setting[Title/Abstract])) OR (Sleep Disorder, Limit Setting[Title/Abstract])) OR (Nocturnal Eating-Drinking Syndrome[Title/Abstract])) OR (Eating-Drinking Syndrome, Nocturnal[Title/Abstract])) OR (Eating-Drinking Syndromes, Nocturnal[Title/Abstract])) OR (Nocturnal Eating Drinking Syndrome[Title/Abstract])) OR (Nocturnal Eating-Drinking Syndromes[Title/Abstract])) OR (Syndrome, Nocturnal Eating-Drinking[Title/Abstract])) OR (Syndromes, Nocturnal Eating-Drinking[Title/Abstract])) OR (Adjustment Sleep Disorder[Title/Abstract])) OR (Adjustment Sleep Disorders[Title/Abstract])) OR (Sleep Disorders, Adjustment[Title/Abstract])) OR (Sleep Disorder, Adjustment[Title/Abstract])) OR (Environmental Sleep Disorder[Title/Abstract])) OR (Environmental Sleep Disorders[Title/Abstract])) OR (Sleep Disorders, Environmental[Title/Abstract])) OR (Sleep Disorder, Environmental[Title/Abstract])) OR ("Dyssomnias"[Mesh]))) OR (((((((((sleep pattern[Title/Abstract]) OR (sleep hygiene[Title/Abstract])) OR (sleep quality[Title/Abstract])) OR (sleep habit[Title/Abstract])) OR (sleep problem[Title/Abstract])) OR (sleep behavior[Title/Abstract])) OR (sleep time[Title/Abstract])) OR (sleep duration[Title/Abstract])) OR (sleep deprivation[Title/Abstract])))

EMBASE

('phenotype, short sleep':ab,ti OR 'phenotypes, short sleep':ab,ti OR 'short sleep phenotypes':ab,ti OR 'sleep phenotypes, short':ab,ti OR 'sleep disorders, intrinsic':ab,ti OR 'intrinsic sleep disorder':ab,ti OR 'sleep disorder, intrinsic':ab,ti OR 'intrinsic sleep disorders':ab,ti OR 'sleep state misperception':ab,ti OR 'sleep state misperceptions':ab,ti OR 'hypersomnia, post-traumatic':ab,ti OR 'hypersomnia, post traumatic':ab,ti OR 'hypersomnias, post-traumatic':ab,ti OR 'post-traumatic hypersomnia':ab,ti OR 'post-traumatic hypersomnias':ab,ti OR 'hypersomnia, posttraumatic':ab,ti OR 'hypersomnias, posttraumatic':ab,ti OR 'posttraumatic hypersomnia':ab,ti OR 'posttraumatic hypersomnias':ab,ti OR 'sleep disorders, circadian rhythm':ab,ti OR 'disturbed nyctohemeral rhythm':ab,ti OR 'disturbed nyctohemeral rhythms':ab,ti OR 'nyctohemeral rhythm, disturbed':ab,ti OR 'nyctohemeral rhythms, disturbed':ab,ti OR 'circadian rhythm sleep disorders':ab,ti OR 'sleep-wake schedule disorders':ab,ti OR 'sleep wake schedule disorders':ab,ti OR 'sleep-wake schedule disorder':ab,ti OR 'sleep-wake cycle disorders':ab,ti OR 'sleep wake cycle disorders':ab,ti

OR 'sleep-wake cycle disorder':ab,ti OR 'shift-work sleep disorder':ab,ti OR 'shift work sleep disorder':ab,ti OR 'shift-work sleep disorders':ab,ti OR 'sleep disorders, shift-work':ab,ti OR 'sleep disorder, shift-work':ab,ti OR 'sleep disorder, shift work':ab,ti OR 'non-24 hour sleep-wake disorder':ab,ti OR 'non 24 hour sleep wake disorder':ab,ti OR 'sleep-wake disorder, non-24 hour':ab,ti OR 'sleep wake disorder, non 24 hour':ab,ti OR 'nonorganic sleep wake cycle disorders':ab,ti OR 'advanced sleep phase syndrome':ab,ti OR 'delayed sleep phase syndrome':ab,ti OR 'delayed sleep-phase syndrome':ab,ti OR 'delayed sleep-phase syndromes':ab,ti OR 'dyssomnias':ab,ti OR 'dyssomnia':ab,ti OR 'sleep disorders, extrinsic':ab,ti OR 'extrinsic sleep disorder':ab,ti OR 'extrinsic sleep disorders':ab,ti OR 'sleep disorder, extrinsic':ab,ti OR 'limit-setting sleep disorder':ab,ti OR 'limit setting sleep disorder':ab,ti OR 'limit-setting sleep disorders':ab,ti OR 'sleep disorders, limit-setting':ab,ti OR 'sleep disorder, limit-setting':ab,ti OR 'sleep disorder, limit setting':ab,ti OR 'nocturnal eating-drinking syndrome':ab,ti OR 'eating-drinking syndrome, nocturnal':ab,ti OR 'eating-drinking syndromes, nocturnal':ab,ti OR 'nocturnal eating drinking syndrome':ab,ti OR 'nocturnal eating-drinking syndromes':ab,ti OR 'syndromes, nocturnal eating-drinking':ab,ti OR 'syndrome, nocturnal eating-drinking':ab,ti OR 'adjustment sleep disorder':ab,ti OR 'adjustment sleep disorders':ab,ti OR 'sleep disorders, adjustment':ab,ti OR 'sleep disorder, adjustment':ab,ti OR 'environmental sleep disorder':ab,ti OR 'environmental sleep disorders':ab,ti OR 'sleep disorders, environmental':ab,ti OR 'sleep disorder, environmental':ab,ti OR 'sleep pattern':ab,ti OR 'sleep hygiene':ab,ti OR 'sleep problem':ab,ti OR 'sleep time':ab,ti OR 'sleep duration':ab,ti OR 'sleep habits':ab,ti OR 'sleep quality':ab,ti OR 'short sleep phenotype':ab,ti OR 'syndromes, short sleeper':ab,ti OR 'syndrome, short sleeper':ab,ti OR 'sleeper syndromes, short':ab,ti OR 'sleeper syndrome, short':ab,ti OR 'short sleeper syndromes':ab,ti OR 'short sleeper syndrome':ab,ti OR 'syndromes, long sleeper':ab,ti OR 'sleeper syndromes, long':ab,ti OR 'long sleeper syndromes':ab,ti OR 'long sleeper syndrome':ab,ti OR 'tachypneas, sleep-related neurogenic':ab,ti OR 'sleep-related neurogenic tachypneas':ab,ti OR 'sleep related neurogenic tachypnea':ab,ti OR 'neurogenic tachypneas, sleep-related':ab,ti OR 'neurogenic tachypnea, sleep-related':ab,ti OR 'sleep-related neurogenic tachypnea':ab,ti OR 'sleep disorder':ab,ti OR 'disorders, sleep':ab,ti OR 'sleep disorders':ab,ti OR 'syndrome, subwakefullness':ab,ti OR 'subwakefullness syndromes':ab,ti OR 'subwakefullness syndrome':ab,ti OR 'wake disorders, sleep':ab,ti OR 'wake disorder, sleep':ab,ti OR 'sleep wake disorder':ab,ti OR 'disorders, sleep wake':ab,ti OR 'disorder, sleep wake':ab,ti OR 'sleep wake disorders':ab,ti OR 'disorder, sleep':ab,ti) AND ('prevalence':ab,ti OR 'epidemiology survey':ab,ti OR 'cross-section study':ab,ti OR 'rate':ab,ti) AND ('adolescents':ab,ti OR 'adolescence':ab,ti OR 'teens':ab,ti OR 'teenagers':ab,ti OR 'youth':ab,ti OR 'youths':ab,ti OR 'students':ab,ti OR 'elementary school students':ab,ti OR 'high school students':ab,ti) AND ('chinese':ab,ti OR 'china':ab,ti OR 'macau':ab,ti OR 'hong kong':ab,ti OR 'taiwan':ab,ti OR 'taiwanese':ab,ti)

Cochrane

#1 (Phenotype, Short Sleep):ab,ti,kw OR (Phenotype, Short Sleep):ab,ti,kw OR (Short Sleep Phenotype):ab,ti,kw OR (Sleep Phenotype, Short):ab,ti,kw OR (Sleep Disorders, Intrinsic):ab,ti,kw OR (Intrinsic Sleep Disorder):ab,ti,kw OR (Sleep Disorder, Intrinsic):ab,ti,kw OR (Intrinsic Sleep Disorders):ab,ti,kw OR (Sleep State Misconception):ab,ti,kw OR (Sleep State Misconception):ab,ti,kw OR (Hypermedia, Post-Traumatic):ab,ti,kw OR (Hypermedia, Post Traumatic):ab,ti,kw OR (Hyperbolas, Post-Traumatic):ab,ti,kw OR (Post-Traumatic

Hypermedia):ab,ti,kw OR (Post-Traumatic Hyperbolas):ab,ti,kw OR (Hypermedia, Post traumatic):ab,ti,kw OR (Hyperbolas, Post traumatic):ab,ti,kw OR (Post traumatic Hypermedia):ab,ti,kw OR (Post traumatic Hyperbolas):ab,ti,kw OR (Sleep Disorders, Circadian Rhythm):ab,ti,kw OR (Disturbed Ephemeral Rhythm):ab,ti,kw OR (Disturbed Ephemeral Rhythms):ab,ti,kw OR (Ephemeral Rhythm, Disturbed):ab,ti,kw OR (Ephemeral Rhythms, Disturbed):ab,ti,kw OR (Circadian Rhythm Sleep Disorders):ab,ti,kw OR (Sleep-Wake Schedule Disorders):ab,ti,kw OR (Sleep Wake Schedule Disorders):ab,ti,kw OR (Sleep-Wake Schedule Disorder):ab,ti,kw OR (Sleep-Wake Cycle Disorders):ab,ti,kw OR (Sleep Wake Cycle Disorders):ab,ti,kw OR (Sleep-Wake Cycle Disorder):ab,ti,kw OR (Shift-Work Sleep Disorder):ab,ti,kw OR (Shift Work Sleep Disorder):ab,ti,kw OR (Shift-Work Sleep Disorders):ab,ti,kw OR (Sleep Disorders, Shift-Work):ab,ti,kw OR (Sleep Disorder, Shift-Work):ab,ti,kw OR (Sleep Disorder, Shift Work):ab,ti,kw OR (Non-24 Hour Sleep-Wake Disorder):ab,ti,kw OR (Non 24 Hour Sleep Wake Disorder):ab,ti,kw OR (Sleep-Wake Disorder, Non-24 Hour):ab,ti,kw OR (Sleep Wake Disorder, Non 24 Hour):ab,ti,kw OR (Non organic Sleep Wake Cycle Disorders):ab,ti,kw OR (Advanced Sleep Phase Syndrome):ab,ti,kw OR (Delayed Sleep Phase Syndrome):ab,ti,kw OR (Delayed Sleep-Phase Syndrome):ab,ti,kw OR (Delayed Sleep-Phase Syndromes):ab,ti,kw OR (Insomnia):ab,ti,kw OR (Insomnia):ab,ti,kw OR (Sleep Disorders, Extrinsic):ab,ti,kw OR (Extrinsic Sleep Disorder):ab,ti,kw OR (Extrinsic Sleep Disorders):ab,ti,kw OR (Sleep Disorder, Extrinsic):ab,ti,kw OR (Limit-Setting Sleep Disorder):ab,ti,kw OR (Limit Setting Sleep Disorder):ab,ti,kw OR (Limit-Setting Sleep Disorders):ab,ti,kw OR (Sleep Disorders, Limit-Setting):ab,ti,kw OR (Sleep Disorder, Limit-Setting):ab,ti,kw OR (Sleep Disorder, Limit Setting):ab,ti,kw OR (Nocturnal Eating-Drinking Syndrome):ab,ti,kw OR (Eating-Drinking Syndrome, Nocturnal):ab,ti,kw OR (Eating-Drinking Syndromes, Nocturnal):ab,ti,kw OR (Nocturnal Eating Drinking Syndrome):ab,ti,kw OR (Nocturnal Eating-Drinking Syndromes):ab,ti,kw OR (Syndromes, Nocturnal Eating-Drinking):ab,ti,kw OR (Syndrome, Nocturnal Eating-Drinking):ab,ti,kw OR (Adjustment Sleep Disorder):ab,ti,kw OR (Adjustment Sleep Disorders):ab,ti,kw OR (Sleep Disorders, Adjustment):ab,ti,kw OR (Sleep Disorder, Adjustment):ab,ti,kw OR (Environmental Sleep Disorder):ab,ti,kw OR (Environmental Sleep Disorders):ab,ti,kw OR (Sleep Disorders, Environmental):ab,ti,kw OR (Sleep Disorder, Environmental):ab,ti,kw OR (Sleep Pattern):ab,ti,kw OR (Sleep Hygiene):ab,ti,kw OR (Sleep Problem):ab,ti,kw OR (Sleep Time):ab,ti,kw OR (Sleep Duration):ab,ti,kw OR (Sleep Habits):ab,ti,kw OR (Sleep Quality):ab,ti,kw OR (Short Sleep Phenotype):ab,ti,kw OR (Syndromes, Short Sleeper):ab,ti,kw OR (Syndrome, Short Sleeper):ab,ti,kw OR (Sleeper Syndromes, Short):ab,ti,kw OR (Sleeper Syndrome, Short):ab,ti,kw OR (Short Sleeper Syndromes):ab,ti,kw OR (Short Sleeper Syndrome):ab,ti,kw OR (Syndromes, Long Sleeper):ab,ti,kw OR (Sleeper Syndromes, Long):ab,ti,kw OR (Long Sleeper Syndromes):ab,ti,kw OR (Long Sleeper Syndrome):ab,ti,kw OR (Cheapness, Sleep-Related Neurological):ab,ti,kw OR (Sleep-Related Neurological Cheapness):ab,ti,kw OR (Sleep Related Neurological Tachycardia):ab,ti,kw OR (Neurological Cheapness, Sleep-Related):ab,ti,kw OR (Neurological Tachycardia, Sleep-Related):ab,ti,kw OR (Sleep-Related Neurological Tachycardia):ab,ti,kw OR (Sleep Disorder):ab,ti,kw OR (Disorders, Sleep):ab,ti,kw OR (Sleep Disorders):ab,ti,kw OR (Syndrome, Wakefulness):ab,ti,kw OR (Wakefulness Syndromes):ab,ti,kw OR (Wakefulness Syndrome):ab,ti,kw OR (Wake Disorders, Sleep):ab,ti,kw OR (Wake Disorder, Sleep):ab,ti,kw OR (Sleep Wake Disorder):ab,ti,kw OR (Disorders, Sleep Wake):ab,ti,kw OR (Disorder, Sleep Wake):ab,ti,kw OR (sleep wake

disorders):ab,ti,kw OR (Disorder, Sleep):ab,ti,kw
2 (prevalence):ab,ti,kw OR (epidemiology survey):ab,ti,kw OR (cross-section study):ab,ti,kw OR (rate):ab,ti,kw
#3 (adolescents):ab,ti,kw OR (adolescence):ab,ti,kw OR (teens):ab,ti,kw OR (teenagers):ab,ti,kw OR (youth):ab,ti,kw OR (youths):ab,ti,kw OR (students):ab,ti,kw OR (elementary school students):ab,ti,kw OR (high school students):ab,ti,kw
#4 (Chinese):ab,ti,kw OR (China):ab,ti,kw OR (Macau):ab,ti,kw OR (Hong Kong):ab,ti,kw OR (Taiwan):ab,ti,kw OR (Taiwanese):ab,ti,kw

Web of Science
#4 AND #3 AND #2 AND #1
#4 TS=(Chinese OR China OR Macau OR Hong Kong OR Taiwan OR Taiwanese)
#3 TS=(adolescents
OR adolescence OR teens OR teenagers OR youth OR youths OR students OR elementary s chool students OR high school students)
#2 TS=(prevalence OR epidemiology survey OR cross-section study OR rate)
#1 TS=(sleep
wake disorders OR Disorder, Sleep Wake OR Disorders, Sleep Wake OR Sleep Wake Disor der OR Wake Disorder, Sleep OR Wake Disorders, Sleep OR Wakefulness Syndrome OR W akefulness Syndromes OR Syndrome, Wakefulness OR Syndromes, Wakefulness OR Sleep Di sorders OR Disorder, Sleep OR Disorders, Sleep OR Sleep Disorder OR Sleep-Related Neuro logical Tachycardia OR Neurological Tachycardia, Sleep-Related OR Neurological Cheapness, Sleep-Related OR Sleep-Related Neurological Cheapness OR Sleep Related Neurological Tac hycardia OR Cheapness, Sleep-Related Neurological OR Tachycardia, Sleep-Related Neurologi cal OR Long Sleeper Syndrome OR Long Sleeper Syndromes OR Sleeper Syndrome, Long OR Sleeper Syndromes, Long OR Syndrome, Long Sleeper OR Syndromes, Long Sleeper O R Short Sleeper Syndrome OR Short Sleeper Syndromes OR Sleeper Syndrome, Short OR S yndrome, Short Sleeper OR Sleeper Syndromes, Short OR Syndromes, Short Sleeper OR Phe notype, Short Sleep OR Short Sleep Phenotype OR Phenotype, Short Sleep OR Short Sleep Phenotype OR Sleep Phenotype, Short OR Sleep Disorders, Intrinsic OR Intrinsic Sleep Diso rder OR Intrinsic Sleep Disorders OR Sleep Disorder, Intrinsic OR Sleep State Misconceptio n OR Sleep State Misconception OR Hypermedia, Post-Traumatic OR Hypermedia, Post Trau matic OR Hyperbolas, Post-Traumatic OR Post-Traumatic Hypermedia OR Post-Traumatic Hy perbolas OR Hypermedia, Post traumatic OR Hyperbolas, Post traumatic OR Post traumatic Hypermedia OR Post traumatic Hyperbolas OR Sleep Disorders, Circadian Rhythm OR Distu rbed Ephemeral Rhythm OR Disturbed Ephemeral Rhythms OR Ephemeral Rhythm, Disturbe d OR Ephemeral Rhythms, Disturbed OR Circadian Rhythm Sleep Disorders OR Sleep-Wake Schedule Disorders OR Sleep Wake Schedule Disorders OR Sleep-Wake Cycle Disorders O R Sleep-Wake Schedule Disorder OR Sleep Wake Cycle Disorders OR Sleep-Wake Cycle Di sorder OR Shift-Work Sleep Disorder OR Shift Work Sleep Disorder OR Shift-Work Sleep Disorders OR Sleep Disorders, Shift-Work OR Sleep Disorder, Shift-Work OR Sleep Disorde r, Shift Work OR Non-24 Hour Sleep-Wake Disorder OR Non 24 Hour Sleep Wake Disorde r OR Sleep-Wake Disorder, Non-24 Hour OR Non organic Sleep Wake Cycle Disorders OR Sleep Wake Disorder, Non 24 Hour OR Advanced Sleep Phase Syndrome OR Delayed Slee

p Phase Syndrome OR Delayed Sleep-Phase Syndrome OR Delayed Sleep-Phase Syndromes OR Insomnia OR Insomnia OR Sleep Disorders, Extrinsic OR Extrinsic Sleep Disorder OR Extrinsic Sleep Disorders OR Sleep Disorder, Extrinsic OR Limit-Setting Sleep Disorder OR Limit Setting Sleep Disorder OR Limit-Setting Sleep Disorders OR Sleep Disorders, Limit-Setting OR Sleep Disorder, Limit-Setting OR Sleep Disorder, Limit Setting OR Nocturnal Eating-Drinking Syndrome OR Eating-Drinking Syndrome, Nocturnal OR Eating-Drinking Syndromes, Nocturnal OR Nocturnal Eating Drinking Syndrome OR Nocturnal Eating-Drinking Syndromes OR Syndrome, Nocturnal Eating-Drinking OR Syndromes, Nocturnal Eating-Drinking OR Adjustment Sleep Disorder OR Adjustment Sleep Disorders OR Sleep Disorders, Adjustment OR Sleep Disorder, Adjustment OR Environmental Sleep Disorder OR Environmental Sleep Disorders OR Sleep Disorders, Environmental OR Sleep Disorder, Environmental OR Sleep Pattern OR Sleep Hygiene OR Sleep Problem OR Sleep Time OR Sleep Duration OR Sleep Habits OR Sleep Quality)

Scopus

(TITLE-ABS-KEY (*sleep AND wake AND disorders) OR TITLE-ABS-KEY (*disorder, AND sleep AND wake) OR TITLE-ABS-KEY (*disorders, AND sleep AND wake) OR TITLE-ABS-KEY (*sleep AND wake AND disorder) OR TITLE-ABS-KEY (*wake AND disorder, AND sleep) OR TITLE-ABS-KEY (*wake AND disorders, AND sleep) OR TITLE-ABS-KEY (*subwakefullness AND syndrome) OR TITLE-ABS-KEY (*subwakefullness AND syndromes) OR TITLE-ABS-KEY (*syndrome, AND subwakefullness) OR TITLE-ABS-KEY (*syndromes, AND subwakefullness) OR TITLE-ABS-KEY (*sleep AND disorders) OR TITLE-ABS-KEY (*disorder, AND sleep) OR TITLE-ABS-KEY (*disorders, AND sleep) OR TITLE-ABS-KEY (*sleep AND disorder) OR TITLE-ABS-KEY (*sleep-related AND neurogenic AND tachypnea) OR TITLE-ABS-KEY (*neurogenic AND tachypnea, AND sleep-related) OR TITLE-ABS-KEY (*neurogenic AND tachypneas, AND sleep-related) OR TITLE-ABS-KEY (*sleep AND related AND neurogenic AND tachypnea) OR TITLE-ABS-KEY (*sleep-related AND neurogenic AND tachypneas) OR TITLE-ABS-KEY (*tachypnea, AND sleep-related AND neurogenic) OR TITLE-ABS-KEY (*tachypneas, AND sleep-related AND neurogenic) OR TITLE-ABS-KEY (*long AND sleeper AND syndrome) OR TITLE-ABS-KEY (*long AND sleeper AND syndromes) OR TITLE-ABS-KEY (*sleeper AND syndrome, AND long) OR TITLE-ABS-KEY (*sleeper AND syndromes, AND long) OR TITLE-ABS-KEY (*syndrome, AND long AND sleeper) OR TITLE-ABS-KEY (*syndromes, AND long AND sleeper) OR TITLE-ABS-KEY (*short AND sleeper AND syndrome) OR TITLE-ABS-KEY (*short AND sleeper AND syndromes) OR TITLE-ABS-KEY (*sleeper AND syndrome, AND short) OR TITLE-ABS-KEY (*sleeper AND syndromes, AND short) OR TITLE-ABS-KEY (*syndrome, AND short AND sleeper) OR TITLE-ABS-KEY (*syndromes, AND short AND sleeper) OR TITLE-ABS-KEY (*short AND sleep AND phenotype) OR TITLE-ABS-KEY (*phenotype, AND short AND sleep) OR TITLE-ABS-KEY (*phenotypes, AND short AND sleep) OR TITLE-ABS-KEY (*short AND sleep AND phenotypes) OR TITLE-ABS-KEY (*sleep AND phenotypes, AND short) OR TITLE-ABS-KEY (*sleep AND disorders,

AND intrinsic) OR TITLE-ABS-KEY (*intrinsic AND sleep AND disorder) OR TITLE-ABS-KEY (*sleep AND disorder, AND intrinsic) OR TITLE-ABS-KEY (*intrinsic AND sleep AND disorders) OR TITLE-ABS-KEY (*sleep AND state AND misperception) OR TITLE-ABS-KEY (*sleep AND state AND misperceptions) OR TITLE-ABS-KEY (*hypersomnia, AND post-traumatic) OR TITLE-ABS-KEY (*hypersomnia, AND post AND traumatic) OR TITLE-ABS-KEY (*hypersomnias, AND post-traumatic) OR TITLE-ABS-KEY (*post-traumatic AND hypersomnia) OR TITLE-ABS-KEY (*post-traumatic AND hypersomnias) OR TITLE-ABS-KEY (*hypersomnia, AND posttraumatic) OR TITLE-ABS-KEY (*hypersomnias, AND posttraumatic) OR TITLE-ABS-KEY (*posttraumatic AND hypersomnia) OR TITLE-ABS-KEY (*posttraumatic AND hypersomnias) OR TITLE-ABS-KEY (*sleep AND disorders, AND circadian AND rhythm) OR TITLE-ABS-KEY (*disturbed AND nyctohemeral AND rhythm) OR TITLE-ABS-KEY (*disturbed AND nyctohemeral AND rhythms) OR TITLE-ABS-KEY (*nyctohemeral AND rhythm, AND disturbed) OR TITLE-ABS-KEY (*nyctohemeral AND rhythms, AND disturbed) OR TITLE-ABS-KEY (*circadian AND rhythm AND sleep AND disorders) OR TITLE-ABS-KEY (*sleep-wake AND schedule AND disorders) OR TITLE-ABS-KEY (*sleep AND wake AND schedule AND disorders) OR TITLE-ABS-KEY (*sleep-wake AND schedule AND disorder) OR TITLE-ABS-KEY (*sleep-wake AND cycle AND disorders) OR TITLE-ABS-KEY (*sleep AND wake AND cycle AND disorders) OR TITLE-ABS-KEY (*sleep-wake AND cycle AND disorder) OR TITLE-ABS-KEY (*shift-work AND sleep AND disorder) OR TITLE-ABS-KEY (*shift AND work AND sleep AND disorder) OR TITLE-ABS-KEY (*shift-work AND sleep AND disorders) OR TITLE-ABS-KEY (*sleep AND disorders, AND shift-work) OR TITLE-ABS-KEY (*sleep AND disorder, AND shift-work) OR TITLE-ABS-KEY (*sleep AND disorder, AND shift AND work) OR TITLE-ABS-KEY (*non-24 AND hour AND sleep-wake AND disorder) OR TITLE-ABS-KEY (*non 24 hour AND sleep AND wake AND disorder) OR TITLE-ABS-KEY (*sleep-wake AND disorder, AND non-24 AND hour) OR TITLE-ABS-KEY (*sleep AND wake AND disorder, AND non 24 hour) OR TITLE-ABS-KEY (*nonorganic AND sleep AND wake AND cycle AND disorders) OR TITLE-ABS-KEY (*advanced AND sleep AND phase AND syndrome) OR TITLE-ABS-KEY (*delayed AND sleep AND phase AND syndrome) OR TITLE-ABS-KEY (*delayed AND sleep-phase AND syndrome) OR TITLE-ABS-KEY (*delayed AND sleep-phase AND syndromes) OR TITLE-ABS-KEY (*dyssomnias) OR TITLE-ABS-KEY (*dyssomnia) OR TITLE-ABS-KEY (*sleep AND disorders, AND extrinsic) OR TITLE-ABS-KEY (*extrinsic AND sleep AND disorder) OR TITLE-ABS-KEY (*extrinsic AND sleep AND disorders) OR TITLE-ABS-KEY (*sleep AND disorder, AND extrinsic) OR TITLE-ABS-KEY (*limit-setting AND sleep AND disorder) OR TITLE-ABS-KEY (*limit AND setting AND sleep AND disorder) OR TITLE-ABS-KEY (*limit-setting AND sleep AND disorders) OR TITLE-ABS-KEY (*sleep AND disorders, AND limit-setting) OR TITLE-ABS-KEY (*sleep AND disorder, AND limit-setting) OR TITLE-ABS-KEY (*sleep AND disorder, AND limit AND setting) OR TITLE-ABS-KEY (*nocturnal AND eating-drinking AND syndrome) OR TITLE-ABS-KEY (*eating-drinking AND syndrome, AND nocturnal) OR

TITLE-ABS-KEY (*eating-drinking AND syndromes, AND nocturnal) OR TITLE-ABS-KEY (*nocturnal AND eating AND drinking AND syndrome) OR TITLE-ABS-KEY (*nocturnal AND eating-drinking AND syndromes) OR TITLE-ABS-KEY (*syndrome, AND nocturnal AND eating-drinking) OR TITLE-ABS-KEY (*syndromes, AND nocturnal AND eating-drinking) OR TITLE-ABS-KEY (*adjustment AND sleep AND disorder) OR TITLE-ABS-KEY (*adjustment AND sleep AND disorders) OR TITLE-ABS-KEY (*sleep AND disorders, AND adjustment) OR TITLE-ABS-KEY (*sleep AND disorder, AND adjustment) OR TITLE-ABS-KEY (*environmental AND sleep AND disorder) OR TITLE-ABS-KEY (*environmental AND sleep AND disorders) OR TITLE-ABS-KEY (*sleep AND disorders, AND environmental) OR TITLE-ABS-KEY (*sleep AND disorder, AND environmental) OR TITLE-ABS-KEY (*sleep AND pattern) OR TITLE-ABS-KEY (*sleep AND hygiene) OR TITLE-ABS-KEY (*sleep AND problem) OR TITLE-ABS-KEY (*sleep AND time) OR TITLE-ABS-KEY (*sleep AND duration) OR TITLE-ABS-KEY (*sleep AND habits) OR TITLE-ABS-KEY (*sleep AND quality)) AND (TITLE-ABS-KEY (*prevalence) OR TITLE-ABS-KEY (*epidemiology AND survey) OR TITLE-ABS-KEY (*cross-section AND study) OR TITLE-ABS-KEY (*rate)) AND (TITLE-ABS-KEY (*adolescents) OR TITLE-ABS-KEY (*adolescence) OR TITLE-ABS-KEY (*teens) OR TITLE-ABS-KEY (*teenagers) OR TITLE-ABS-KEY (*youth) OR TITLE-ABS-KEY (*youths) OR TITLE-ABS-KEY (*students) OR TITLE-ABS-KEY (*elementary AND school AND students) OR TITLE-ABS-KEY (*high AND school AND students)) AND (TITLE-ABS-KEY (*chinese) OR TITLE-ABS-KEY (*china) OR TITLE-ABS-KEY (*macau) OR TITLE-ABS-KEY (*hong AND kong) OR TITLE-ABS-KEY (*taiwan) OR TITLE-ABS-KEY (*taiwanese))

PsycINFO

((abstract: (adolescent)) OR (abstract: (teens)) OR (abstract: (teenagers)) OR (abstract: (youth)) OR (abstract: (youths)) OR (abstract: (students))) AND ((abstract: (China)) OR (abstract: (Chinese)) OR (abstract: (Macau)) OR (abstract: (Hong Kong)) OR (abstract: (Taiwan)) OR (abstract: (Taiwanese))) AND ((abstract: (prevalence)) OR (abstract: (epidemiology survey)) OR (abstract: (cross-section study)) OR (abstract: (rate))) AND ((title: (Sleep Disorders)) OR (title: (Sleep Wake Disorders)) OR (title: (Intrinsic Sleep Disorders)) OR (title: (Circadian Rhythm Sleep Disorders)) OR (title: (Sleep Quality)) OR (title: (Sleep Problem)) OR (title: (Dyssomnia)) OR (title: (Sleep Pattern)) OR (title: (Sleep Habits)) OR (title: (Sleep Hygiene)) OR (title: (Sleep Time)) OR (title: (Sleep Duration)) OR (title: (Disorder, Sleep Wake)) OR (title: (Disorders, Sleep Wake)) OR (title: (Sleep Wake Disorder)) OR (title: (Wake Disorder, Sleep)) OR (title: (Wake Disorders, Sleep)) OR (title: (Subwakefullness Syndrome)) OR (title: (Subwakefullness Syndromes)) OR (title: (Syndrome, Subwakefullness)) OR (title: (Syndromes, Subwakefullness)) OR (title: (Sleep Disorders)) OR (title: (Disorder, Sleep)) OR (title: (Disorders, Sleep)) OR (title: (Sleep Disorder)) OR (title: (Sleep-Related Neurogenic Tachypnea)) OR (title: (Neurogenic Tachypnea, Sleep-Related)) OR (title: (Neurogenic Tachypneas, Sleep-Related)) OR (title: (Sleep Related Neurogenic Tachypnea)) OR (title: (Sleep-Related Neurogenic Tachypneas)) OR (title: (Tachypnea, Sleep-Related Neurogenic)) OR (title: (Tachypneas, Sleep-Related Neurogenic)) OR (title: (Long Sleeper Syndrome)) OR (title: (Long Sleeper

Syndromes)) OR (title: (Sleeper Syndrome, Long)) OR (title: (Sleeper Syndromes, Long)) OR (title: (Syndrome, Long Sleeper)) OR (title: (Syndromes, Long Sleeper)) OR (title: (Short Sleeper Syndrome)) OR (title: (Short Sleeper Syndromes)) OR (title: (Sleeper Syndrome, Short)) OR (title: (Sleeper Syndromes, Short)) OR (title: (Syndrome, Short Sleeper)) OR (title: (Syndromes, Short Sleeper)) OR (title: (Short Sleep Phenotype)) OR (title: (Phenotype, Short Sleep)) OR (title: (Phenotypes, Short Sleep)) OR (title: (Short Sleep Phenotypes)) OR (title: (Sleep Phenotypes, Short)) OR (title: (Sleep Disorders, Intrinsic)) OR (title: (Sleep State Misperception)) OR (title: (Sleep State Misperceptions)) OR (title: (Hypersomnia, Post-Traumatic)) OR (title: (Hypersomnia, Post Traumatic)) OR (title: (Hypersomnias, Post-Traumatic)) OR (title: (Post-Traumatic Hypersomnia)) OR (title: (Post-Traumatic Hypersomnias)) OR (title: (Hypersomnia, Posttraumatic)) OR (title: (Hypersomnias, Posttraumatic)) OR (title: (Posttraumatic Hypersomnia)) OR (title: (Posttraumatic Hypersomnia)) OR (title: (Posttraumatic Hypersomnia)) OR (title: (Disturbed Nyctohemeral Rhythm)) OR (title: (Nyctohemeral Rhythm, Disturbed)) OR (title: (Sleep-Wake Schedule Disorders)) OR (title: (Sleep-Wake Cycle Disorders)) OR (title: (Shift-Work Sleep Disorder)) OR (title: (Sleep Disorders, Shift-Work)) OR (title: (Non-24 Hour Sleep-Wake Disorder)) OR (title: (Sleep-Wake Disorder, Non-24 Hour)) OR (title: (Nonorganic Sleep Wake Cycle Disorders)) OR (title: (Advanced Sleep Phase Syndrome)) OR (title: (Delayed Sleep Phase Syndrome)) OR (title: (Sleep Disorders, Extrinsic)) OR (title: (Extrinsic Sleep Disorders)) OR (title: (Limit-Setting Sleep Disorder)) OR (title: (Sleep Disorders, Limit-Setting)) OR (title: (Nocturnal Eating-Drinking Syndrome)) OR (title: (Eating-Drinking Syndrome, Nocturnal)) OR (title: (Adjustment Sleep Disorders)) OR (title: (Environmental Sleep Disorders)) OR (title: (Sleep Disorder, Environmental)) OR (title: (Sleep Disorder, Adjustment)))

SInoMed(Chinese)

("青少年"[常用字段:智能] OR "青年"[常用字段:智能] OR "少年"[常用字段:智能] OR "小学生"[常用字段:智能] OR "初中生"[常用字段:智能] OR "高中生"[常用字段:智能] OR "学生"[常用字段:智能] OR "中学生"[常用字段:智能] OR "职校生"[常用字段:智能]) AND ("横断面研究"[常用字段:智能] OR "发病率"[常用字段:智能] OR "报告率"[常用字段:智能] OR "发生率"[常用字段:智能] OR "检出率"[常用字段:智能] OR "患病率"[常用字段:智能] OR "患病率调查"[常用字段:智能] OR "发病率调查"[常用字段:智能] OR "流行率调查"[常用字段:智能]) AND (("睡眠时长"[常用字段:智能] OR "睡眠习惯"[常用字段:智能] OR "睡眠时型"[常用字段:智能] OR "睡眠卫生"[常用字段:智能] OR "睡眠剥夺"[常用字段:智能]) OR ("睡眠障碍"[常用字段:智能] OR "睡眠觉醒障碍"[常用字段:智能] OR "睡眠异常"[常用字段:智能] OR "睡眠障碍,内源性"[常用字段:智能] OR "睡眠障碍，昼夜节律性"[常用字段:智能] OR "入睡和睡眠障碍"[常用字段:智能] OR "失眠"[常用字段:智能] OR "睡眠质量"[常用字段:智能] OR "睡眠问题"[常用字段:智能]))

Appendix 2 AHRQ- Score of Quality Assessment

Item	4		5		6		7		N	Overall
	n	Response Rate(%)	n	Response Rate(%)	n	Response Rate(%)	n	Response Rate(%)		Response Rate(100%)
#1	13	100	23	100	18	100	9	100	63	100
#2	13	100	23	100	18	100	9	100	63	100
#3	2	15.38	15	65	15	83	9	100	41	65
#4	0	0	0	0	0	0	0	0	0	0
#5	0	0	0	0	0	0	0	0	0	0
#6	1	7.69	5	22	16	89	9	100	31	49
#7	11	84.62	23	100	18	100	9	100	61	97
#8	0	0	0	0	3	17	5	56	8	13
#9	0	0	2	9	2	11	3	33	7	11
#10	13	100	23	100	18	100	9	100	63	100
#11	0	0	0	0	0	0	0	0	0	0

n: Number of studies in score 4/5/6/7 responded to each item separately.
N: Total number of studies responded to each item

Items 1--11:
1) Define the source of information (survey, record review)
2) List inclusion and exclusion criteria for exposed and unexposed subjects (cases and controls) or refer to previous publications
3) Indicate time period used for identifying patients
4) Indicate whether or not subjects were consecutive if not population-based
5) Indicate if evaluators of subjective components of study were masked to other aspects of the status of the participants
6) Describe any assessments undertaken for quality assurance purposes (e.g., test/retest of primary outcome measurements)

7) Explain any patient exclusions from analysis
8) Describe how confounding was assessed and/or controlled.
9) If applicable, explain how missing data were handled in the analysis
10) Summarize patient response rates and completeness of data collection
11) Clarify what follow-up, if any, was expected and the percentage of patients for which incomplete data or follow-up was obtained

PRISMA 2009 Checklist

Section/topic	#	Checklist item	Reported on page #
**TITLE**			
Title	1	Identify the report as a systematic review, meta-analysis, or both.	1
**ABSTRACT**			
Structured summary	2	Provide a structured summary including, as applicable: background; objectives; data sources; study eligibility criteria, participants, and interventions; study appraisal and synthesis methods; results; limitations; conclusions and implications of key findings; systematic review registration number.	2
**INTRODUCTION**			
Rationale	3	Describe the rationale for the review in the context of what is already known.	3
Objectives	4	Provide an explicit statement of questions being addressed with reference to participants, interventions, comparisons, outcomes, and study design (PICOS).	3
**METHODS**			
Protocol and registration	5	Indicate if a review protocol exists, if and where it can be accessed (e.g., Web address), and, if available, provide registration information including registration number.	N/A
Eligibility criteria	6	Specify study characteristics (e.g., PICOS, length of follow-up) and report characteristics (e.g., years considered, language, publication status) used as criteria for eligibility, giving rationale.	4
Information sources	7	Describe all information sources (e.g., databases with dates of coverage, contact with study authors to identify additional studies) in the search and date last searched.	4
Search	8	Present full electronic search strategy for at least one database, including any limits used, such that it could be repeated.	4
Study selection	9	State the process for selecting studies (i.e., screening, eligibility, included in systematic review, and, if applicable, included in the meta-analysis).	4
Data collection process	10	Describe method of data extraction from reports (e.g., piloted forms, independently, in duplicate) and any processes for obtaining and confirming data from investigators.	5
Data items	11	List and define all variables for which data were sought (e.g., PICOS, funding sources) and any assumptions and simplifications made.	5
Risk of bias in individual studies	12	Describe methods used for assessing risk of bias of individual studies (including specification of whether this was done at the study or outcome level), and how this information is to be used in any data synthesis.	5
Summary measures	13	State the principal summary measures (e.g., risk ratio, difference in means).	5,6
Synthesis of results	14	Describe the methods of handling data and combining results of studies, if done, including measures of consistency (e.g., I^2) for each meta-analysis.	5,6

PRISMA 2009 Checklist

Section/topic	#	Checklist item	Reported on page #
Risk of bias across studies	15	Specify any assessment of risk of bias that may affect the cumulative evidence (e.g., publication bias, selective reporting within studies).	6
Additional analyses	16	Describe methods of additional analyses (e.g., sensitivity or subgroup analyses, meta-regression), if done, indicating which were pre-specified.	5,6
**RESULTS**			
Study selection	17	Give numbers of studies screened, assessed for eligibility, and included in the review, with reasons for exclusions at each stage, ideally with a flow diagram.	6
Study characteristics	18	For each study, present characteristics for which data were extracted (e.g., study size, PICOS, follow-up period) and provide the citations.	6-11
Risk of bias within studies	19	Present data on risk of bias of each study and, if available, any outcome level assessment (see item 12).	7
Results of individual studies	20	For all outcomes considered (benefits or harms), present, for each study: (a) simple summary data for each intervention group (b) effect estimates and confidence intervals, ideally with a forest plot.	12
Synthesis of results	21	Present results of each meta-analysis done, including confidence intervals and measures of consistency.	12
Risk of bias across studies	22	Present results of any assessment of risk of bias across studies (see Item 15).	17
Additional analysis	23	Give results of additional analyses, if done (e.g., sensitivity or subgroup analyses, meta-regression [see Item 16]).	12-16
**DISCUSSION**			
Summary of evidence	24	Summarize the main findings including the strength of evidence for each main outcome; consider their relevance to key groups (e.g., healthcare providers, users, and policy makers).	17,18
Limitations	25	Discuss limitations at study and outcome level (e.g., risk of bias), and at review-level (e.g., incomplete retrieval of identified research, reporting bias).	18,19
Conclusions	26	Provide a general interpretation of the results in the context of other evidence, and implications for future research.	19
**FUNDING**			
Funding	27	Describe sources of funding for the systematic review and other support (e.g., supply of data); role of funders for the systematic review.	N/A

From: Moher D, Liberati A, Tetzlaff J, Altman DG, The PRISMA Group (2009). Preferred Reporting Items for Systematic Reviews and Meta-Analyses: The PRISMA Statement. PLoS Med 6(7): e1000097. doi:10.1371/journal.pmed1000097

For more information, visit: **www.prisma-statement.org**.
